# Antibiotics, cancer risk and oncologic treatment efficacy: a practical review of the literature

**DOI:** 10.3332/ecancer.2020.1106

**Published:** 2020-09-21

**Authors:** Moises S Martins Lopes, Larissa M Machado, Pedro A Ismael Amaral Silva, Angel A Tome Uchiyama, Cheng T Yen, Eliza D Ricardo, Taciana S Mutao, Jefferson R Pimenta, Denis S Shimba, Rodrigo M Hanriot, Renata D Peixoto

**Affiliations:** 1 Hospital Alemão Oswaldo Cruz, São Paulo, Brazil; 2 Centro Paulista de Oncologia (Grupo Oncoclínicas), São Paulo, Brazil

**Keywords:** antibiotics, microbiome, cancer, immunotherapy, chemotherapy

## Abstract

Antibiotics have been extensively used to treat infectious diseases over the past century and have largely contributed to increased life expectancy over time. However, antibiotic use can impose profound and protracted changes to the diversity of the microbial ecosystem, affecting the composition of up to 30% of the bacterial species in the gut microbiome. By modifying human microbiota composition, antibiotics alter the action of several oncologic drugs, potentially leading to decreased efficacy and increased toxicities. Whether antibiotics interfere with cancer therapies or even increase the risk of cancer development has been under investigation, and no randomised trials have been conducted so far. The aim of the current review is to describe the possible effects of antibiotic therapies on different oncologic treatments, especially immunotherapies, and to explore the link between previous antibiotics use and the development of cancer.

## Introduction

More than 100 years ago, infectious diseases accounted for the highest morbidity and mortality worldwide [1]. The discovery of penicillin in 1928 by Alexander Fleming marked the beginning of the antibiotic revolution, which led to its wide use since 1945 and changed the landscape of infectious disease in many countries [2]. During decades, we have been witnessing a continuous growth of antibiotics use, and only from 2000 to 2015, antibiotic consumption increased by 65% [3].

Antibiotic therapy has produced unquestionable advances in the management of patients, especially those with cancer, a population with an intrinsically higher risk of bacterial infection as a result of malignancy or treatment-related immune suppression. Amongst oncologic patients, antibiotic consumption has also escalated over time, and whilst contributing to reduced mortality from several infections, it certainly impacts negatively on commensal bacterial species which live on or in individuals, also known as microbiota [4]. These organisms and their genes, metabolites and interactions with one another, as well as with their host collectively, represent the microbiome [4].

Changes in the microbiome as a result of antibiotic treatment, especially gut microbiota dysbiosis, may result in the dysregulation of host immune homeostasis and increased susceptibility to several diseases, including cancer [5]. Whether antibiotics interfere with cancer therapies or even increase the risk of cancer development has been under investigation, and no randomised trials have been conducted so far.

The aim of the current review is to describe the possible effects of antibiotic therapies on different oncologic treatments, especially immunotherapies, and to explore the link between previous antibiotics use and the development of cancer. For this purpose, a PubMed search was conducted, and the articles exploring the effects of antibiotics on human microbiome as well as those including the potential risk of cancer development with antibiotics’ use and their influence on cancer therapeutics were selected and revised.

## Influence of antibiotic therapy on the microbiome

The microbiome has been denominated the second genome and is composed by bacteria, archaea, fungi and viruses, reaching a much larger number of cells and genes than those derived from the human gametes [6]. Typically, there are three categories of gut bacteria based on their functions in the host: symbionts, with mutual benefit to the host; conditioned pathobionts, normally harmless but producing disease due to fortuitous events that affect the host; and pathobionts, disease-causing organisms [7–9]. The human microbiomes are divided into four major phyla: Firmicutes (65%), Bacteroidetes (16%), Actinobacteria (9%) and Proteobacteria (5%) [10].

This complex ecosystem integrates gut microbiota and has a profound effect on all aspects of human health, such as protecting the host against pathogenic bacteria, promoting digestion, absorption, drug and carcinogens’ metabolism, regulating energy metabolism [11–17] and exerting a key role in inflammation pathways and the regulation of innate and adaptive immunological processes [18]. A healthy child’s microbiome increases in diversity in parallel with the maturation of the immune system until the age of two or three, resembling an adult microbiome [19]. Indeed, aberrant neonatal microbiota composition is associated with disease during childhood and later in life [20]. The proper function of the gut microbiome depends on a delicate homeostasis that can be disturbed by the action of many factors, such as age, diet composition, antibiotic therapies, lifestyle and physical activity, exerting an impact on gut microbiome and equilibrium [21, 22]. The disruption of this equilibrium is known as dysbiosis.

Antibiotics have been extensively used to treat infectious diseases over the past century and largely contributed to increased life expectancy over time. However, high doses and frequent use of antibiotics, particularly against anaerobes (such as vancomycin), can disrupt and destabilise the orderly bowel microbiome with complex repercussions to the tumour–host–microbe interface [23]. Antibiotics impose profound and protracted changes to the diversity of the host–microbial ecosystem, affecting the composition of up to 30% of the bacterial species in the gut microbiome, consequently leading to the loss of microbial functions that are protective for the host [24]. By modifying human microbiota composition, antibiotics alter the action of several oncologic drugs, potentially leading to decreased efficacy and increased toxicities [25].

Drug metabolism by intestinal microorganisms has been well recognised since the 1960s’[26]. However, we are still lacking a complete map of microbiota–host–drug interactions in cancer therapy [25]. Indeed, chemotherapeutic agents may exacerbate dysbiosis instead of ameliorating it, with potentially serious implications for drug tolerability [25]. In addition, oncologic drugs are known to induce changes in the diversity of the mucosal and faecal microbiota through altered biliary excretion and secondary metabolism [25].

Immunomodulation is also an important mechanism which occurs in the gut microbiota and leads to several treatment‐induced immune and inflammatory responses [25]. *Lactobacillus* and segmented filamentous bacteria, for instance, mediate the accumulation of type 17 T-helper (TH17) cell and type 1 T-helper (TH1) cell responses [27]. As a consequence, pathologic species might predominate, leading to deleterious diarrhoea and/or colitis [25]. This is the main physiopathology mechanism involved in the case of immunotherapy, in which the direct effect of antibiotics could induce selective pressure within the host microbiome and transform microbiota by the downregulation of major histocompatibility complex class I/II genes and impaired effector T-cell responses, which are implicated in reduced responsiveness to immunotherapy [25, 28, 29].

It has also been recently suggested that some species of bacteria provide intrinsic immune-modulating properties [29]. *Bacteroidetes* phylum, for example, appears to have a protective effect against checkpoint inhibitor-induced colitis [30]. Overall, CTLA-4 (cytotoxic T-lymphocyte-associated protein 4) inhibition requires the presence of specific bacteria to work, whereas anti-PD-1 drugs seem to interact only partially with gut microbiota [31].

On the other hand, immunotherapy can increase the number of potentially dangerous bacterial species. Specifically, it may increase the number of *Clostridiales* whilst decreasing the number of *Bacteroidales* and *Burkholderiales,* which could affect the response to cancer therapy [28]. With all these examples, it is not difficult to understand how antibiotic-induced changes in the microbiota may affect cancer treatment efficacy and toxicity.

## Cancer risk with antibiotics

Relevant publications have raised the hypothesis that certain drugs are associated with carcinogenesis [32, 33] and that the regular use of antibacterial drugs may be associated with cancer development [34]. According to a recent meta-analysis of 25 observational case–control or cohort studies, there is moderate evidence that the prolonged or excessive use of antibiotics during a person’s life is associated with a slight increased risk of various types of cancers [35]. Besides, a nested case–control study for 15 common malignancies revealed that a recurrent exposure to certain antibiotics frequently used in the community may be associated with cancer risk in specific organ sites [35, 36]. Since antibiotics have no known direct carcinogenic effect, the main hypothesis for the increased cancer risk focuses on their influence on the composition of the human microbiome, which involves the bacteria that compose the microbiota, their genes, metabolites and interactions with one another, as well as with their host collectively, including the immune system [4, 37].

In the aforementioned meta-analysis, the primary outcome was the risk of developing cancer in ever versus non-antibiotic users amongst 7,947,270 individuals. On the primary analysis of overall cancer incidence, the previous exposure to antibiotics increased the risk of cancer by 18% (odds ratio (OR): 1.18, *p* < 0.001), which was particularly increased for the following primary tumours: lung cancer (OR 1.29, *p* = 0.02), renal cell carcinoma (OR 1.28, *p* = 0.001), pancreatic cancer (OR 1.28, *p* = 0.019), lymphomas (OR 1.31, *p* < 0.001) and multiple myeloma (OR 1.36, 95% CI 1.18–1.56, *p* < 0.001). Higher risks were found in individuals with either a long duration of antibiotic exposure or higher doses [35]. In addition, the antibiotic classes with the strongest significant association with cancer development were beta-lactams, macrolides and quinolones [35].

Aside from intestinal microbiota, the association of local microbiota and some cancer types has been investigated, linking local dysbiosis and carcinogenesis [38–41]. For lung cancer, chronic inflammation linked to altered lung microbiota could explain local carcinogenesis [38]. Although the lungs were once considered sterile organs, a low-density, the diversified microbial ecosystem is currently known to be present in bronchoalveolar lavage fluid, sputum and lung tissues. Furthermore, several bacteria species have been shown to be enriched in lung cancer patients compared with healthy individuals [38]. Modifications in lung microbiota induced by antibiotics might explain the higher incidence of lung cancer amongst the users of antibiotics in the aforementioned meta-analysis [35].

Similar data were published for genitourinary and pancreatobiliary cancer as well as for lymphomas [39–42]. The urinary tract is also a host of an array of bacteria in healthy individuals, and the changes in this microbiome have been observed in certain urologic disorders, including cancers [39, 40]. Similarly, the liver, biliary tract and pancreas may be susceptible to altered local microbiota. In addition, those tissues are constantly exposed to the gut microbiome via blood flow through the portal vein, inferring that gut microbiome may influence hepatobiliary-pancreatic diseases through this gut–liver axis [41]. For lymphomas, the close interaction between the immune system and the intestinal microbiota might explain the development of some hematologic malignancies, such as mucosal-associated lymphoid tissue lymphoma [42].

Colonic microbiota is more commonly composed of anaerobic bacteria. Therefore, differently from other tumour types, whose relationship is more intense with intravenous antibiotics, sporadic colorectal cancer has a stronger association with oral anti-anaerobic antibiotics. Meanwhile, colorectal cancer tissues are enriched with polymicrobial invasive biofilms, particularly on right-sided tumours. Thus, the greater antibiotic impact on the proximal colon may reflect the disruption of biofilm formation, which has been linked with pro-carcinogenesis [43–50]. The most common class of antibiotics related to colon cancer development is anti-anaerobic, and the main one is penicillin [48, 50].

On the contrary, the risk of rectal cancer has been curiously reduced with the use of tetracyclines [49, 51]. Several studies report their anti-inflammatory and potential antineoplastic effect [52, 53]. Possible biological mechanisms contributing to diminished neoplastic risk from antibiotic exposure include the inhibition of mitochondrial protein synthesis, matrix metalloproteinases and/or angiogenesis. In addition, antibiotics can eradicate pathogens (such as those causing sexually transmitted diseases) that may contribute to malignant transformation [53, 54].

## Antibiotics and cancer treatment

In the latter years, immune checkpoint inhibitors (ICI) became incontestable breakthrough advance in cancer treatment, particularly for solid tumours. There is increasing evidence that gut dysbiosis due to antibiotics exposure has an interference with immune responsiveness [55].

In a multicentre prospective study, a total of 196 patients (119 of them with non-small cell lung cancer (NSCLC), 38 with melanoma and 39 with other tumour types) were treated with ICI [56] and had their OS and response evaluated according to the prior or concurrent use of antibiotics. Respiratory tract infection was the most common indication for antibiotics exposure and possibly relates to the majority of patients having NSCLC. Prior antibiotic therapy was significantly associated with worse median OS compared to no prior antibiotics (2 versus 26 months; HR 7.4, *p* < 0.001). There was also a much higher likelihood of primary disease refractory to ICI therapy (81 versus 44%, respectively; *p* < 0.001). Interestingly, concomitant antibiotic therapy was not associated with worse outcomes. The negative impact of prior antibiotic use with ICI was observed across all tumour types and was independent of disease burden and performance status [56].

A retrospective study with 121 renal cell carcinoma and 239 NSCLC patients, who received ICI from two academic institutions in France and USA, evaluated oncologic outcomes according to antibiotics’ use within 30 days of beginning ICI versus no antibiotics [57]. Although only 64 patients had received any antibiotic (most commonly β-lactam or quinolones for pneumonia or urinary tract infections), an increased risk of primary progressive disease was associated with antibiotics’ use (75 versus 22%, *p* < 0.01). In addition, both progression-free survival (PFS) (median 1.9 versus 7.4 months, HR 3.1, and *p* < 0.01) and OS (median 17.3 versus 30.6 months, HR 3.5, and *p* = 0.03) were shorter amongst antibiotics’ users, independently of the tumour type [57]. To investigate the difference in antibiotic timing, the authors conducted a subgroup analysis for patients who had received antibiotics 60 days before ICI administration. They were able to demonstrate that the impact of antibiotics 2 months before ICI was not as relevant as within 1 month before ICI. Although those data still need further confirmation, it has been hypothesised that antibiotics shift microbiota composition temporally, and its recovery increases with time.

Pooled data from the phase II POPLAR and phase III OAK trials with 1,515 NSCLC patients randomised to either atezolizumab or docetaxel revealed that one-quarter of them had received prior antibiotics. For those who were in the atezolizumab arms, antibiotics’ use was associated with decreased median OS (8.5 versus 14.1 months, HR 1.32, 95% CI: 1.06–1.63, and *p* = 0.01). On the other hand, within the docetaxel population, there was no association between the use of antibiotics and OS [58]. Despite those results suggest that the use of antibiotics in patients with metastatic NSCLC is associated with poor outcome and may influence the efficacy of ICI, those analyses were all unplanned and retrospective in nature.

In Switzerland, 218 patients with advanced NSCLC had their outcomes evaluated according to prior use (15.1% of the cohort) or not of antibiotics within 2 months before ICI (59). Compared to non-users, prior antibiotic use was significantly associated with lower response rate (18.2% versus 28.3%, *p* = 0.02), shorter PFS (median 1.4 versus 5.5 months, HR 2.22, and *p* < 0.01) and worse OS (median 1.8 versus 15.4 months, HR 2.61, and *p* < 0.01). In a sensitivity analysis, the use of ATB either during ICI treatment or within 1 month after ICI discontinuation had no deleterious effect on outcomes.

Similar results were described amongst melanoma patients. In a retrospective analysis of a group of 568 stage III or IV melanoma patients, the use of antibiotics up to 90 days before the beginning of ICI led to an impairment in the 2-year melanoma-specific mortality rate (47 versus 37.4%, HR 1.95, and *p* = 0.03). The incidence of immune-mediated colitis was also higher in antibiotics’ exposed patients, who also required more use of steroids (9.8% in exposed versus 4.6% in non-exposed patients, HR 2.14, and *p* = 0.03) [60]. A possible explanation to the increased immune-mediated colitis is that dysbiosis caused by antibiotics may reduce the population of microorganisms that promote regulatory T-cells in mucosa with the consequent growth of pro-inflammation bacterial [61, 62]. A small prospective study with 26 metastatic melanoma patients treated with the anti-CTLA4 ipilimumab showed that patients with gut microbiota rich in Bacterioidetes and microorganisms known to be involved in regulatory T-cell differentiation developed no immune-related colitis [63].

The way the antibiotic is administered may also impact on outcomes when ICI is concerned. In a retrospective analysis with 291 metastatic cancer patients (melanoma, renal cell carcinoma or NSCLC) who were treated with ICI, antibiotic users during ICI therapy had a significant reduction on median PFS (3.1 versus 6.3 months, HR 1.56, and *p* = 0.003) and OS (10.4 versus 21.7 months, HR 1.69, *p* =0.002) [64]. In addition, when the authors looked at the antibiotic use itself, they found that when only a single course of antibiotics was administrated, no detrimental effect was seen on PFS or OS, whereas patients who had received the cumulative courses of antibiotics (duration of therapy longer than 7 days and/or more than one intravenous or oral antibiotic or sequential antibiotic use for multiple sources of infection) had significantly worse PFS (median PFS 2.8 months, HR 2.625, and *p* = 0.026) and OS (median OS, 6.3 months, HR 1.904, and *p* = 0.009) [64]. Similarly, a retrospective analysis involving 157 patients with metastatic NSCLC treated with ICI found that the length of antibiotic therapy in relation to ICI mattered, with multiple or prolonged cycles of antibiotics correlating negatively with PFS and OS [65]. Other retrospective analyses also pointed out to the negative effects of antibiotics on ICI outcomes [66, 67].

Meta-analyses also tried to address the question whether antibiotics interfere with ICI efficacy. The first meta-analysis to evaluate the association between the use of antibiotics and ICI in cancer patients included 19 eligible studies with 2,740 patients. The authors showed that the use of antibiotics was associated with worse OS (HR = 2.37 and *p* < 0.001), without heterogeneity. Furthermore, antibiotic exposure also significantly reduced PFS in cancer patients treated with ICIs (HR = 1.84 and *p* < 0.001). An unfavourable impact on outcomes was also demonstrated irrespective of the time of the use of antibiotics and type of cancer. Subgroup analysis on the type of ICI revealed a negative impact on all drugs and combinations [68].

In another meta-analysis including 18 studies with multiple tumour types and 2889 patients, 826 of whom had been exposed to antibiotics, and OS with ICI was prolonged amongst those without antibiotic exposure (HR 1.92 and *p* < 0.0001) (69). The effect of antibiotics on OS was more pronounced amongst patients exposed to antibiotics 42 days prior to ICI treatment (HR 3.43 and *p* < 0.0001), indicating that the timing of antibiotic exposure in relation to ICI is also relevant [69]. Other meta-analysis that pointed out to the same detrimental effects of antibiotics on ICI therapy was performed with 5,745 NSCLC patients from 23 studies [70]. Both PFS and OS were shorter amongst antibiotics’ users, and median OS was reduced on average by 6.7 months (70). In line with the other studies, the authors of this meta-analysis also noted an influence of the time window of exposure to antibiotics on the ICI treatment effect, with a greater impact when antibiotics were taken shortly before or after ICI initiation.

Nonetheless, not all studies found a negative impact on the use of antibiotics in the efficacy of ICI. A small retrospective study with 74 NSCLC patients treated with nivolumab could not find a detrimental effect of the use of antibiotics [71]. However, only 15 (20.3%) patients had been exposed to antibiotic medication in the 3 months before the first nivolumab injection or during treatment. Therefore, the small number of patients could have influenced the negative results in this study. Most data reported so far indicate that ICI treatment may be influenced by the use of antibiotics.

Antibiotics are frequently prescribed during the course of chemotherapy although their effect on cancer treatment outcomes is poorly described, and most of the scarce data come from animal models. It has been well known that chemotherapy can cause profound dysbiosis and affect multiple metabolic pathways [72, 73]. Meanwhile, the majority of antibiotics prescribed in this setting have broad-spectrum activity with a high potential to alter the normal microbiota [74].

A study conducted in mice suggested that antibiotic administration disrupted the gut microbiota, which contributed to the reduction of antitumour efficacy of 5-fluorouracil [75]. In a similar fashion, cyclophosphamide has been shown to alter the microbiota composition in mice small bowel and induce the translocation of selected species of Gram-positive bacteria into secondary lymphoid organs, where the generation of a pathogenic subset of T-helper 17 cells and memory T-helper 1 immune responses occurred [27]. Interestingly, germ-free or antibiotics-treated tumour-bearing mice had a reduction in T-helper 17 responses, whereas their tumours were resistant to cyclophosphamide [27]. In this mouse model, the reduction in the antitumour effect of cyclophosphamide was more pronounced after exposure to Gram-positive spectrum antibiotics, as opposed to antibiotics with Gram-negative spectrum.

Another study conducted in mice showed the disruption of the microbiota impaired the response of tumours to platinum chemotherapy [76]. In antibiotics-treated or germ-free mice, tumour-infiltrating myeloid-derived cells had a less response to therapy, resulting in the deficient production of reactive oxygen species through Toll-like receptors and lower cytotoxicity after platinum agents. Thus, the authors concluded that optimal responses to cancer therapy in mice require an intact commensal microbiota that modulates myeloid-derived cell functions in the tumour microenvironment.

To investigate the impact of antibiotic treatment on antineoplastic treatment outcomes in humans, a German group identified 800 patients treated with a cyclophosphamide containing first-line therapy for chronic lymphocytic leukaemia (CLL) as well as 122 patients treated with a cisplatin containing regimen for relapsed lymphoma (RL). Potential associations between anti-Gram-positive antibiotic treatment and patient outcome were retrospectively analysed [77]. For both CLL and RL cohorts, treatment with anti-Gram-positive antibiotics was significantly associated with worse response rates, PFS and OS, indicating a potential negative impact of anti-Gram-positive antibiotics on the anticancer activity of cyclophosphamide and cisplatin. The prospective studies are urgently needed to confirm those findings. Data on the deleterious effect of microbiota disruption by early exposure to broad-spectrum antibiotics also exist amongst 621 patients undergoing allogeneic stem cell transplantation (ASCT). Antibiotic administration before ASCT was significantly associated with a higher transplant-related mortality compared to post-ASCT or no antibiotics (34% versus 21% versus 7%, respectively) [78].

Similar findings have been reported amongst patients with solid malignancies. In a recent retrospective study, the impact of antibiotic therapy on treatment outcomes following curative-intent chemotherapy and RT in patients with locally advanced head and neck cancer (LAHNC) was analysed. Patients who had received antibiotics progressed significantly earlier compared with patients in the non-antibiotic group (median PFS 147.8 months versus not reached). Overall survival (OS) was also significantly lower in patients who had received antibiotics (71.9 versus 132 months, *p* = 0.0007). Antibiotics were an independent prognostic factor for PFS and OS in this study. Furthermore, the use of more than two antibiotics was related to an even worse negative impact [74].

Not only efficacy but also chemotherapy-induced toxicity may be altered with antimicrobial agents. A recent analysis from the MPACT trial revealed that patients with metastatic pancreatic adenocarcinoma treated with gemcitabine had an increased rate of gemcitabine-associated toxicity during and after antibiotic therapy. This observation comes in line with preclinical evidence that intratumour bacteria metabolise gemcitabine to an inactive form [79].

The interaction of radiotherapy with anti-neoplastic drugs is already reasonably well known, with positive interactions, such as sensitisation (concurrent and supra-additive), protective interactions (sub-additive, inhibitory or antagonistic) or inert interactions, both for anti-neoplastic efficacy and side effects, as well as anachronistic interactions, the so-called ‘recall effect’ [80].

More recently, the interaction between nutritional supplements, more specifically anti-oxidants (pentoxifylline, tocopherol and ascorbic acid in high doses, glucosamines, beta-carotenes and so on) have brought conflicting data, either by potentially reducing effectiveness or not showing any interference with chemotherapy or radiation therapy. As such, the current recommendation is a temporary suspension of those agents for irradiation treatment, unless the antioxidant is considered essential [81].

When antibiotics are concerned, the modifications of the intestinal microbiome induced by those drugs may lead to greater toxicity when concomitant or sequential pelvic radiotherapy is used [82, 83]. Animal studies have shown that vancomycin use, an antibiotic that acts against Gram-positive bacteria and is restricted to the intestinal tract, promotes an increase in the antitumour potential of radiotherapy despite greater toxicity [84]. However, scarce information is available on a possible interaction between antibiotics and radiation therapy in humans.

For LAHNC, a retrospective analysis of 272 patients found a negative association with antibiotics when used between 1 week before the start and 2 weeks after the end of the combined chemoradiation therapy. Interestingly, 45.6% of patients had received antibiotics during the course of their anticancer treatment. Of the 272 patients in the total sample, 233 of them (85%) received induction chemotherapy (cisplatin or carboplatin with 5-fluorouracil) followed by chemoradiotherapy (with cisplatin or carboplatin), 12 were treated with induction chemotherapy followed by radiation alone, only 6 with chemoradiotherapy alone, 17 with surgery followed by chemoradiotherapy, 3 with radiotherapy alone and only one with radiotherapy associated with cetuximab. A negative impact was observed on PFS, disease-specific survival (DSS) and overall survival (OS), with HRs of 1.98 (*p* = 0.001), 1.95 (*p* = 0.004) and 1.85 (*p* = 0.001), respectively. The use of two or more courses of antibiotic therapy had the greatest negative impact on PFS, DSS and OS, whereas the use of probiotics had a reducing effect on this risk. Given the negative influence of antibiotics on outcomes, the authors recommended avoiding the use of broad-spectrum antibiotics whenever possible, especially for prophylactic purposes (74). A prospective randomised phase II trial with 95 LAHCNC patients evaluated the role of prophylactic antibiotic therapy with amoxicillin/clavulanic acid versus standard care without prophylaxis during chemoradiotherapy (85). The authors found no difference in the primary endpoint (reduction in pneumonia) although a lower rate of hospitalisation and febrile episodes were reported in the prophylactic group, with a better impact on the final cost and quality of life. However, there is no long-term evaluation of PFS, DFS or OS (85,86), and those results are eagerly awaited.

Briefly, the concomitant use of antibiotics and radiotherapy has yet little evidence of safety or interaction. However, given the few clinical studies, the recommendation is to minimise antimicrobial use as much as possible, adopting preventive measures and limiting prophylactic use.

## What could be done to minimise the potential risks of antibiotics

The composition of gut microbiome is known to be influenced by genetics, lifestyle, immune conditions of the patient, comorbidities and previous treatment including the previous use of antibiotics. All of these factors may have a favourable influence or not on the cancer treatment of patients [87].

As previously mentioned, several studies have shown that the use of antibiotics close to or concurrent with immunotherapy is associated with reduced OS and response to treatment [57, 88], whereas the interaction of antibiotics with other oncologic therapies is also being investigated. It has been a consensus that the use of broad-spectrum antibiotics should be avoided during the use of immunotherapy whenever possible. In addition, antibiotics should be prescribed only when properly indicated. On the other hand, one may argue that the use of antibiotics could turn a bad microbiome, for example, a microbiome rich in bacteria that promote immunosuppression through the expansion of FoxP3 Tregs, into a proper gut microbiome [89]. Furthermore, the clinical studies are needed to better understand the role of antibiotics and their interactions with the microbiome.

Since antibiotics kill bacteria based on broad features, such as Gram-positive or negative staining, they do not seem the best alternative to eliminate specific pathogens from the microbiome. Probably, a better approach would be to modulate the existing commensal community via prebiotics or dietary changes to favour the expansion of beneficial bacteria or, more specifically, to identify metabolic pathways utilised by bacteria and target them [90].

A thought-provoking study published in 2014 showed that short-term consumption of diets composed entirely of animal products, which means a diet rich in meats, eggs and cheeses or plant products, mainly grains, legumes, fruits and vegetables, alters microbial community structure and overwhelms interindividual differences in microbial gene expression. The animal-based diet increased the abundance of bile-tolerant microorganisms (*Alistipes, Bilophila* and *Bacteroides*) and decreased the levels of Firmicutes that metabolise dietary plant polysaccharides (*Roseburia, Eubacterium rectale* and *Ruminococcus bromii*). The animal-based diet supports a link between dietary fat, bile acids and the outgrowth of microorganisms capable of triggering inflammatory bowel disease [91]. The process of T-cell differentiation utilises the short-chain fatty acid butyrate, which allows one to hypothesise the possibility of bypass gut microbiota by the use of short-chain fatty acid supplements [92–94]. These studies reveal the importance that the research on the microbiome needs to be comprehensive, including interactions with metabolism and immunology in order to develop specific intervention strategies to target the host microbiota [95].

Another potential approach to alter gut microbiome is faecal microbiota transplantation (FMT). Despite the encouraging results of FMT in the treatment of refractory *Clostridium difficile* diarrhoea, it requires a consideration of several key factors such as the definition of a favourable microbiota, the possibility of delivering immune-regulatory bacteria and the potential to transfer disease-promoting bacteria (89,96). A small pilot study of four FMT post-hematopoietic stem cell transplants with steroid-refractory graft-versus-host disease (GVHD) showed a remarkable resolution of GVHD in 75% of patients [97]. However, the development of invasive infection caused by multidrug-resistant organisms has been reported in two patients receiving FMT treatment, and one of the patients died. Furthermore, the clinical investigation should focus on both FMT-induced adverse events and its oncologic efficacy [98]. Currently, many studies are being conducted, and their results are expected to provide a better understanding of the intestinal microbiome as a therapeutic target in cancer treatment (Table 1).

## Conclusion

The composition of gut microbiome is influenced by genetics, lifestyle, immune conditions of the patient, comorbidities and previous treatment including the previous use of antibiotics. Several studies have linked dysbiosis to carcinogenesis, whereas others have shown that the use of antibiotics close to or concurrent with cancer therapy may be associated with worse outcomes. Therefore, the use of antibiotics should be avoided whenever possible, and physician education on the possible harms of antibiotics is urgently needed.

## Conflicts of interest

The authors declare no conflicts of interest.

## Funding source

There was no financial funding for this manuscript.

## Figures and Tables

**Table 1.** Current clinical trials with interventions in the gut microbiota

Fecal microbiota transplantation	Probiotics/prebiotics	Diet
NCT 03214289 NCT 04163289 NCT 04014413 NCT 03819803 NCT 02269150 NCT 02770326 NCT 04264975 NCT 04285424 NCT 04269850 NCT 03353402 NCT 03720392 NCT 04203017 NCT 03812705 NCT 03148743 NCT 04116775 NCT 04303286 NCT 03838601 NCT 03686202 NCT 03341143 NCT 04130763	NCT 04193904 NCT 03934827 NCT 03775850 NCT 03595683 NCT 03637803 NCT 03686202 NCT 04208958 NCT 03358511 NCT 03870607 NCT 02544685	NCT 02843425 NCT 03700437 NCT 03679260 NCT 03340935 NCT 03162289 NCT 02710721 NCT 00936364 NCT 03454282 NCT 03087903

Source: https://clinicaltrials.gov/
